# Clinically Significant Dysregulation of *hsa-miR-30d-5p* and *hsa-let-7b* Expression in Patients with Surgically Resected Non-Small Cell Lung Cancer

**Published:** 2018

**Authors:** Sayed Mostafa Hosseini, Bahram Mohammad Soltani, Mahmoud Tavallaei, Seyed Javad Mowla, Elham Tafsiri, Abouzar Bagheri, Hamid Reza Khorram Khorshid

**Affiliations:** 1. Department of Genetics, Faculty of Biological Sciences, Tarbiat Modares University, Tehran, Iran; 2. Human Genetic Research Center, Baqiyatallah University of Medical Sciences, Tehran, Iran; 3. Department of Molecular Medicine, Biotechnology Research Center, Pasteur Institute of Iran, Tehran, Iran; 4. Department of Clinical Biochemistry and Genetics, Molecular and Cell Biology Research Center, Faculty of Medicine, Mazandaran University of Medical Sciences, Sari, Iran; 5. Genetic Research Centre, University of Social Welfare and Rehabilitation Sciences, Tehran, Iran

**Keywords:** Lung cancer, MicroRNAs, Tumor markers

## Abstract

**Background:**

The cyclin E2 (CYCE2) is an important regulator in the progression and development of NSCLC, and its ectopic expression promoted the proliferation, invasion, and migration in several tumors, including Non-Small Cell Lung Cancer (NSCLC). However, the upregulation of CYCE2 in NSCLC cells suggested that it has a key role in tumorigenicity. In addition, the RAS family proteins as oncoproteins were activated in many major tumor types and its suitability as the therapeutic target in NSCLC was proposed. Considering the crucial role of microRNAs, it was hypothesized that altered expression of *hsa-miR-30d-5p* and *hsa-let-7b* might provide a reliable diagnostic tumor marker for diagnosis of NSCLC.

**Method:**

Real-time RT-PCR approach could evaluate the expression alteration of *hsa-miR-30d-5p* and *hsa-let-7b* and it was related to the surgically resected tissue of 24 lung cancer patients and 10 non-cancerous patients. The miRNAs expression was associated with clinicopathological features of the patients.

**Results:**

*Hsa-miR-30d* showed a significant downregulation (p=0.0382) in resected tissue of NSCLC patients compared with control group. Its expression level could differentiate different stages of malignancies from each other. The ROC curve analysis gave it an AUC=0.73 (p=0.037) which was a good score as a reliable biomarker. In contrast, *hsa-let-7b* was significantly overexpressed in tumor samples (p=0.03). Interestingly, our findings revealed a significant association of *hsa-let-7b* in adenocarcinoma tumors, compared to Squamous Cell Carcinomas (SCC) (p<0.05). Also, analysis of ROC curve of *hsa-let-7b* (AUC=0.74, p-value=0.042) suggests that it could be as a suitable biomarker for NSCLC.

**Conclusion:**

Together, these results suggest a possible tumor suppressor role for *hsa-miR-30d* in lung tumor progression and initiation. Moreover, upregulation of *hsa-let-7b* was associated with the tumor type.

## Introduction

Lung cancer is the most common reason of cancer-related mortality for patients’ suffer from cancer in Iran. Owing to life style change, air pollution and increasing tobacco consumption, incidence of lung cancer is growing and it is one of the five common cancer types in Iran^1^.

Non-Small-Cell Lung Cancer (NSCLC) is responsible for about 80–85% of all cases of lung cancer^2^. Late diagnosis of lung cancer is one of the key causes for high mortality^3^. Moreover, the prognosis of patients with end-stage of NSCLC remains unfavorable. By means of presently existing methods, more than around ∼75% of lung tumor are diagnosed at untreatable stages^2,4,5^.

MicroRNAs (miRNA) are endogenous short class of small non-coding RNAs (18–24 nucleotides) which fine-tuning gene expression dosage through targeting mRNAs^6^. MiRNAs regulate an extensive array of biological processes, such as cell proliferation, adhesion, apoptosis, cell death and differentiation^7^.

The *let-7* family, as founding member of miRNAs, is considered as tumor suppressors *via* negatively regulating RAS oncogenes in NSCLC^8^, with downregulated expression correlating with poor prognosis. Aberrant expression of the family members can however promote the reprogramming of these normal processes and motivate cell proliferation and tumor metastasis^9,10^. The *let-7* family was previously evaluated as lung reprogramming-related miRNAs, with aberrant expression levels which promoted NSCLC carcinogenesis.

Several reports indicated that *hsa-hsa-miR-30d* is negatively regulated in both NSCLCs^11^. *hsa-miR-30d* is located at 134804876 *bp* to 134804945 *bp* on chromosome 8q24.22. It belongs to the *has-miR-30* family including *hsa-miR-30a-f* that keeps a sequence highly conserved between species.

Yao *et al* found that *hsa-miR-30d* was upregulated and involved in metastasis process *via* targeting GN-AI2 in hepatocellular carcinoma^12^. Extensive research has shown that *hsa-miR-30d* was deregulated and functions as a tumor suppressor in many tumors, including prostate cancer^13–15^, hepatocellular carcinoma^12^ and medulloblastoma^16^.

Accordingly, miRNA alteration investigation could be used to classify tumors based on their stage of malignancies. miRNAs profile analysis, only or in combination with traditional methods, potentially have the ability to increase chance of diagnosis, prognosis, identifying effectiveness predictive biomarkers and finding more effective molecular targets for novel therapeutic procedures^5,15^. However, the clinical pathologies feature the role of *hsa-let-7b* and *hsa-miR-30d* in the tumorgenesis of NSCLC.

Considering the ectopic expression of cyclin E2 (CYCE2, Gene ID: 9134) in lung tumor tissues and its role in cell cycle G1/S transition and proliferation, it was hypothesized in this study that the expression of *hsa-miR-30d-5p* which targets cyclin E2 might be potentially applied as a tumor marker in NSCLC. In addition, one of the signal transduction pathways related to NSCLC are members of the RAS GTPase family, which contain multiple putative *let-7* binding sites. Using bioinformatics approaches, a putative RAS-targeting microRNA (*hsa-let-7b*) was predicted, and then the expression levels of *hsa-miR-30d* and *hsa-let-7b* in NSCLC tumor samples versus non-tumor tissues were evaluated. Moreover, an attempt was made to investigate whether expression levels in their expression alteration were associated with the clinicopathological features of tumor including staging, smoking status, and type of lung tumor.

## Materials and Methods

### Study design and clinical samples

In this study, the sample included 24 patients with surgically resected NSCLC and 10 matched distant noncancerous tissues from Baqiyatallah Hospital, Tehran, obtained between January 2014 and May 2016. All the patients included in our study had to meet the following criteria: (i) patients must be associated with the diagnosis of NSCLC and (ii) gold standard techniques, comprising lung biopsy samples and imaging procedures, were applied to confirm the histopathological features and tumor stages of NSCLC patients. The exclusion criteria were: (i) none of the patients has ever received radiotherapy or chemotherapy; (ii) there are no significant differences in age, gender and smoking status; and (iii) patients without sufficient data. Ten healthy controls were selected including smokers and non-smokers but with no history of pulmonary diseases as a control group, including 2 Benign Pulmonary Nodule (BPN) patients and 8 Chronic Obstructive Pulmonary Disease (COPD).

All patients had undergone flexible bronchoscopy, improved by rapid on-site evaluation performed by an experienced pathologist, at the bronchoscopy unit. The age of the patients was 37–80 years old (mean, 59.29 years) who were 52 male and 16 female. For tissue sample collection, upon removal of the surgical specimens, the tissues were immediately transported to the Pathology Laboratory and the samples were placed in a cryovial, snap-frozen liquid nitrogen for 30 *min*, and stored at −80°*C* until use.

All samples were evaluated immunohistochemically for the expression of two major markers that have been promoted for the classification of NSCLCs. Expression of thyroid transcription factor-1 (TTF-1) was assessed in alveolar type cells and in bronchiolar cells, and its expression is often retained in adenocarcinomas derived from these cell types. Furthermore, p63 immuno-staining was performed as a means of discerning squamous differentiation in NSCLC in Baqiyatallah Pathology Laboratory. Written informed consent was achieved and the Ethical and Scientific Committees of Baqiyatallah University of Medical Sciences approved this study. After surgery elimination, all resected tissue specimens were immediately immersed in RNAlater liquid buffer and kept at −80°*C* until use^17^. Smoking history, data of tumor histology, and staging were obtained from patients. The NSCLC patients included 14 adenocarcinomas and 10 SCC, and 9 stage I, 9 stage II, 6 stage III as determined according to WHO classification and the International Union against Cancer staging system. The majority of patients (15/24, 62.5%) were smokers. All the necessary information of patients and healthy controls is provided in table 1.

**Table 1.** Demographic and clinical features of surgically resected NSCLC patients

**Case**	**Age**	**Sex**	**Smoker**	**Histology**	**Stage group**	**Lymph node**
**1**	62	m	No	Adeno	III	Positive
**2**	53	m	No	Adeno	III	Positive
**3**	62	m	Yes	Adeno	II	Negative
**4**	61	m	Yes	SCC	II	Positive
**5**	54	f	No	Adeno	II	Positive
**6**	50	m	No	SCC	II	Positive
**7**	80	m	Yes	Adeno	III	Negative
**8**	65	m	Yes	SCC	I	Negative
**9**	56	m	Yes	SCC	II	Negative
**10**	54	f	Na	Adeno	II	Positive
**11**	62	m	Yes	SCC	III	Positive
**12**	60	m	Yes	SCC	III	Positive
**13**	64	m	Yes	SCC	I	Negative
**14**	61	m	Yes	SCC	I	Negative
**15**	55	f	No	Adeno	I	Negative
**16**	37	m	No	Adeno	I	Positive
**17**	60	f	Yes	Adeno	III	Positive
**18**	76	m	Yes	Adeno	II	Negative
**19**	62	f	No	SCC	I	Positive
**20**	52	m	Yes	Adeno	II	Negative
**21**	57	m	Yes	Adeno	I	Negative
**22**	58	m	Yes	Adeno	II	Negative
**23**	53	f	Yes	Adeno	I	Negative
**24**	69	m	No	SCC	I	Negative

m: male, f: female. Smoking history; ≥20 pack years. SCC: small cell cancer; adeno; adenocarcinoma.

### RNA extraction

Total RNA from the lung tissue specimens was extracted *via* TRIzol reagent (Ambion) as formerly described^18^. The pellet of extracted RNA was dissolved in 15 *μl* RNA storage buffer (Invitrogen) and kept at −80°*C* until use. The RNA yield and A 260/280 ratio were calculated by a NanoDrop spectrophotometer (Thermo Fisher Scientific, Waltham, USA), then 25 fmol of an exogenous synthetic miRNA, UniSP6 (Exiqon, Denmark) as an external control for normalization of sample to-sample differences in RNA isolation process was spiked into each tissue specimen^19^.

### RNA polyadenylation, cDNA synthesis and quantitation of miRNA expression by RT-qPCR

Before complimentary DNA synthesis, the samples were treated with RNase-free DNase to remove possible traces of genomic DNA. cDNA (Complimentary DNA) synthesis was then performed on 1 *μg* total RNA based on the poly A tailing method. Initially, a poly A tail was added to the extracted RNAs by incubating 1 *μg* of RNA 2 *μl* of 10 × buffer, 0.5 *μl* of poly A polymerase enzyme, 2 *μl* of ATP, 0.5 *μl* of RNase inhibitor (Thermo, Fisher Scientific, Waltham, USA), and DEPC-treated water at 37°*C* for 10 *min*. Then, for cDNA synthesis, 2 *μl* of polyadenylated RNA was mixed with 2 *μl* of 5 × buffer, 0.5 *μl* of reverse transcriptase (RT) enzyme, 0.5 *μl* of specific primers, and 0.5 *μl* of RNase inhibitor (Ribolock). The mixture was incubated in a thermal cycler at 42°*C* for 60 *min* followed by incubation at 85°*C* for 1 *min*, to heat-in-activate the reverse transcriptase enzyme.

Real-time quantitative PCR (RT-qPCR) was performed using 1 *μl* cDNA product, 0.5 *μl* of specific forward primer, 0.5 *μl* of universal reverse primer (ParsGenome, Iran), 10 *μl* of HOT FIREPol EvaGreen qPCR Mix (Solis BioDyne, Estonia) and 8 *μl* of nuclease-free water (CinnaClon, Iran). The *has-mir-16-5p* and U6 were used as an internal control. Real time qPCR was performed to measure expressions of mi-RNAs by using a Step-one plus system (Applied Biosystems, USA) with the following conditions: 95°*C* for 15 *min*, followed by 40 cycles of 95°*C* for 15 *s*, 61°*C* for 25 *s*, and 72°*C* for 20 *s*. All RT-qPCR amplifications were performed in duplicate, and a negative control was included for each reaction for quality control.

### Statistical analysis

In order to do statistical analysis, firstly, Kolmogorov-Smirnov normality test (KS test) was done to survey the normal distribution of the samples. RT-qPCR data analyses were performed with REST 2009 software (Qiagen, Hilden, Germany) and GraphPad Prism 6 software (GraphPad Software Inc., SanDiego, USA). The results are showed as the mean±standard error of the mean (SEM) from at least three independent trials. Alterations between two groups were analyzed using parametric equations to determine the significance of the observed differences between different groups. All p-values shown were one sided, and a p-value of <0.05 was considered statistically significant. The Receiver Operating Characteristic (ROC) was employed to interpret the optimal sensitivity and specificity levels at which to discriminate normal individuals from NSCLC patients from non-tumor control, and corresponding thresholds were calculated for each miRNA.

## Results

### Real-time quantitative PCR analysis and direct DNA sequencing

All sample types were NSCLC tumor type, from which 15 were high grade (II/III), and 9 were low grade. Total RNA was extracted and RT-qPCR was performed via miR-specific primers and outer primer (Table 2) which amplified ∼70 base pair products on all samples and the validity of the PCR products were confirmed by agarose gel electrophoresis and direct sequencing.

**Table 2.** Primer sequences used in RT-qPCR analysis

**Name**	**Forward**	**Reverse**
***hsa-miR-30d***	TGTAAACATCCCCGACTGGA	GCGAGCACAGAATTAATACGAC
***hsa-let-7b***	TGAGGTAGTAGGTTGTGT	GCGAGCACAGAATTAATACGAC
**U6**	TTTCGCAAGGATGACACGC	GCGAGCACAGAATTAATACGAC
***hsa-miR-16-5p***	TAGCAGCACGTAAATATT	GCGAGCACAGAATTAATACGAC
**Anchored oligo (dT) primer**	GCGAGCACAGAATTAATACGACTCACTATAGG (32bp) (T)12VN^*^	

* V= G, A, C; N= G, A, T, C

All data were normalized to the average expression of *has-mir-16* and U6, as internal microRNA control. There were no non-specific products or primer-dimer peaks in melt-curve analysis (data not shown).

### *Hsa-miR-30d* is significantly downregulated in NSCLC tissues

**Figure 1.**
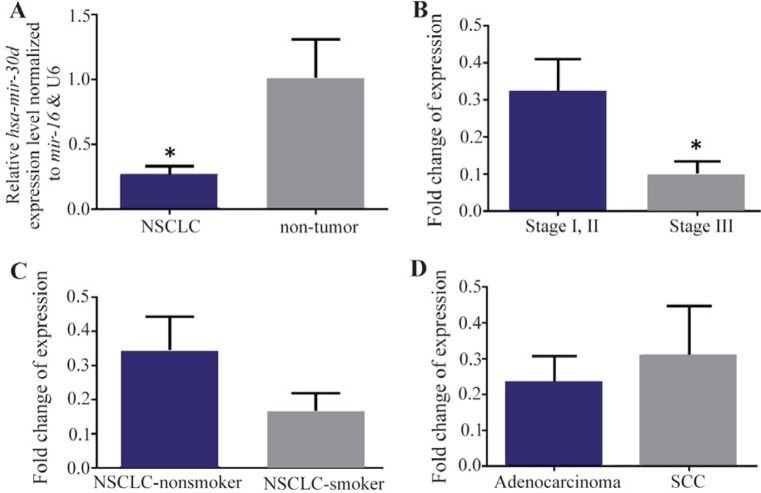
A) RT-qPCR analysis shows the mean values of relative *hsa-miR-30d* expression in Non-Small Cell Lung Cancer (NSCLC) and nontumor controls, with confidence intervals as the error bars. Note that the expression of *hsa-miR-30d* was significantly lower in 24 NSCLC tissues than that in the corresponding nontumor (p= 0.0382). Relative expression levels of *hsa-miR-30d* are demonstrated for different stages, B) and smoking status groups, C) of NSCLC patients. Note that the observed differences in expression were not statistically significant. D) A similar comparison in lung adenocarcinoma *vs*. Squamous Cell Carcinoma (SCC) samples. P-values of <0.05 were considered statistically significant.

Our data showed that there was a significant down-regulation of *hsa-miR-30d* expression levels in NSCLC samples compared to those of their nontumor controls (p<0.0382) (Figure 1A). The patients were separated in two groups including the ones in stages I/II and stage III, and the expression level of *hsa-miR-30d* significantly decreased in stage III tumors in comparison to that of tumor samples with stage I/II (p<0.0420) (Figure 1B). The levels of *hsa-miR-30d* expression in NSCLC-smoker group were higher than those in NSCLC-nonsmoker (Figure 1C). Nevertheless, this alteration was also not statistically significant. Similarity, our data showed no statistically significant association between expression levels of *hsa-miR-30d* in adenocarcinoma in comparison to SCC, which may have been due to the small sample size (24 samples of NSCLC and 10 samples of non-tumors) (Figure 1D).

### *Hsa-let-7b* is significantly upregulated in NSCLC tissues

**Figure 2.**
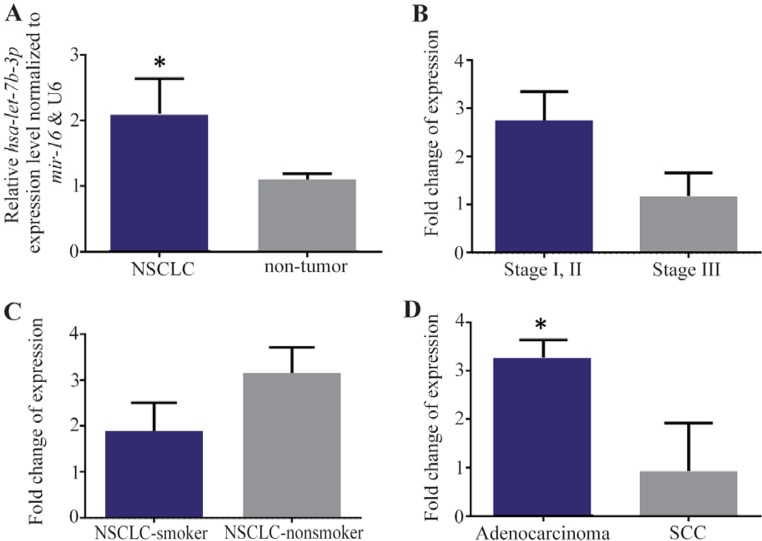
A) RT-qPCR analysis shows the mean values of relative *hsa-let-7b* expression in NSCLC and nontumor controls, with confidence intervals as the error bars. Note that the expression of *hsa-let-7b* was significantly upper in 24 NSCLC tissues vs. nontumor (p= 0.03). Relative expression alterations of *hsa-let-7b* are demonstrated for different stages, B) and smoking status groups, C) of NSCLC patients. D) A similar comparison in lung adenocarcinoma *vs*. SCC samples. P-values of <0.05 were considered statistically significant.

A significant upregulation of *hsa-let-7b* expression alterations was observed in tumor samples compared to those of unpaired nontumor controls (p=0.0382) (Figure 2A). The expression alterations of *hsa-let-7b* were not statistically associated with stages (Figure 2B) and smoking status (Figure 2C). Interestingly, there was a significant upregulation of *hsa-let-7b* in lung adenocarcinomas, compared to the SCC (p=0.02, Figure 2D).

### The specificity and sensitivity of *hsa-miR-30d* and *hsa-let-7b* expression levels in discrimination tumor vs. non-tumor samples

**Figure 3.**
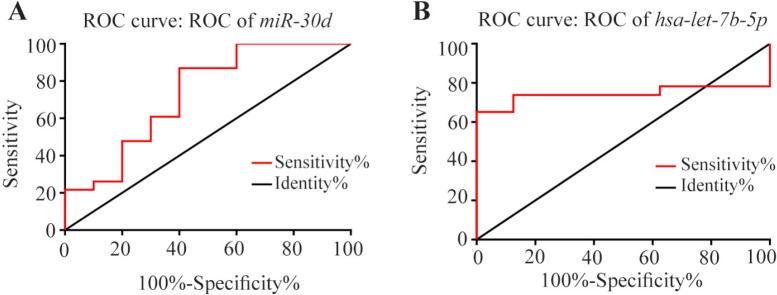
Receiver Operating Characteristic (ROC) curve analysis of the specificity and sensitivity of *hsa-miR-30d* (A) and *hsa-let-7b* (B) expression in discriminating between NSCLC and nontumor samples. The areas under the curve were 73 and 74%, respectively, which suggests that both miRNAs may be potentially tumor markers for NSCLC diagnosis. * Represents p<0.05.

ROC curve analyses were conducted to estimate the sensitivity and specificity of the *hsa-miR-30d* and *hsa-let-7b* expression levels could discriminate tumor/non-tumor states. As it is evident in figure 3, total area under the curves (AUCs) of *hsa-miR-30d* and *hsa-let-7b* were 73 % (CI=0.5264 to 0.9344, p=0.037) and 74% (CI=0.5724 to 0.9167, p=0.042), respectively.

## Discussion

Recently, remarkable improvements have been developed in the discovery of new biomarkers associated with variations at the molecular level of lung cancer. The researches have demonstrated significant clinical relation in different areas such as diagnosis, response to drugs and tumor classification. Many studies have been concentrated on evaluation expression of key microRNAs involved in NSCLC development, progression and manners of tumor in different stages^20,21^.

To investigate the expression alteration of *hsa-miR-30d* in NSCLC patients, *hsa-miR-30d* expression was evaluated in various NSCLC samples and obvious downregulation expression differences in tumor as nontumor specimens were found. In addition, *hsa-miR-30d* and *hsa-let-7b* were expressed in all tumor and nontumor samples (Figures 1 and 2). A significant downregulation of *hsa-miR-30d* was observed in NSCLC resected tissue, suggesting that *hsa-miR-30d* acts as a potential tumor-suppressor in NSCLC (Figure 2). Moreover, a significant expression alteration of *hsa-miR-30d* in stage III NSCLC tissues was found compared to stage I/II. The noticeable reduced expression of *hsa-miR-30d* was found in high-grade tumor, suggesting a dual role of the *hsa-miR-30d* in invasion and initiation of NSCLC.

Le *et al* have reported that *hsa-miR-30d* was overexpressed in pre-operative lung carcinoma patients and paired 10 day post-operative patients in whom its levels were significantly related with survival and its expression level showed that it could be a potentially novel non-invasive biomarker for diagnosis of lung cancer^22^.

Our data are consistent with those of Markou et al’s^23^, which revealed that expression levels of this miRNA were significantly downregulated in NSCLC tissues than in non-cancerous tissues. On the other hand, our results are in contrast with those in a study by Kobayashi *et al*^15^, in which upregulation of *hsa-miR-30d* was shown in prostate cancer. In a previous study, *hsa-miR-30d* has been already shown to be downregulated in thyroid carcinogenesis via targeting polycomb protein enhancer of zeste 2 (EZH2)^24,25^ and in squamous cell lung carcinoma^23^. Moreover, *hsa-miR-30d* expression has revealed to be decreased in some malignancies including ovarian cancer cells *via* Transforming Growth Factor beta1 (TGF-beta1) and pancreatic cancer^25,26^. Recently, *hsa-miR-30d* was found downregulated and it functions as a tumor suppressor by directly targeting the CCNE2 in NSCLC.

One reasonable description for the contradictory reports on miRNA expression in similar cancer types could be that they were the result of applying patients with different epigenetic alteration in genomic context with miscellaneous types, grades or stages of malignancy. Furthermore, *has-miR-30d* has an especial seed sequence that has been demonstrated to bind to several critical genes which have essential role in Epithelial Mesenchymal Transition (EMT) as a key process for the initiation of cancer^26^. Smoking status in patients with NSCLC is related with particular alterations to the epigenomic and genomic landscapes of lung cancer^27^. Therefore, a noticeable underexpression level of *hsa-miR-30d* was seen in smokers of NSCLC, which is in agreement with those in a report written by Vucic *et al*^28^.

*Hsa-let-7b* is one of the most commonly evaluated miRNA in human cancers, gastric cancer^29–31^, melanomas^32^ and lung cancer^33,34^. As mentioned above, a significant upregulation of *hsa-let-7b* expression in NSCLC was found compared to that in their nontumor samples. Accordingly, ozcan *et al* reported that several members of *let-7* cluster had a key role in colorectal carcinoma through Microtubule-associated Tumor Suppressor 1 (MTUS1)^35^. Our findings are in accordance with those of Edmonds *et al*^36^ which showed a significant increase of *hsa-let-7b* in stage I lung adenocarcinomas relapse patients and it appears to be involved in patients with NSCLC. Furthermore, Fassina *et al* have shown upregulation of *hsa-let-7b* in other *let-7* families in adenocarcinoma in resected tumor specimens^33^. In agreement with our findings, over-expression of *hsa-let-7b* in tissue of NSCLC patients is significantly correlated with type of NSCLC, suggesting that expression of *hsa-let-7b* might be useful as a prognostic marker. On the other hand, several studies showed downregulation of the *let-7b* in tumor cell lines^37^ or tumor tissue when compared to their non-tumor^38^.

It has been reported that overexpression of *hsa-let-7b* leads to decreased expression of Insulin-Like Growth Factor receptor 1 (IGF1R) at the post-transcriptional level introducing it as a tumor suppressor in multiple myeloma^39^.

Differentially expressed *hsa-let-7b* was identified when smokers to nonsmokers were compared in NSCLC. Remarkably, the expression levels of both mi-RNAs were considerably lower in the tissue of the smoking NSCLC group than in the nonsmoking control group. The *hsa-let-7b* is a member of the *let-7* family which regulates various cellular processes in cancers and is involved in the growth and proliferation through a potential role in angiogenesis^38^.

The incongruence between these studies and ours led us to apply different methodologies and in addition, not to discriminate between different grades and stages of tumors. Furthermore, one of the possible reasons that can be explained is the conflicting role of *let-7b*, in which the stromal content of tumor microenvironment specimens such as Cancer-Associated Fibroblasts (CAF) can affect the consequence of expression results^40^. One of the limitations of our study was the small sample size.

Our results provide evidence that miRNA expression distinguishes not only tumor tissue from normal tissue but also specific miRNAs for histopathological subtypes of NSCLC. Furthermore, using ROC curve analysis, *has-hsa-miR-30d* and *hsa-let-7b* were found to be potentially good tumor biomarkers to distinguish between NSCLC and non-cancer. Also, the expression level of *hsa-miR-30d* varied significantly in different stages. Remarkably, the pattern of expression in different stages was almost similar for *hsa-let-7b-5p*.

## Conclusion

Taken together, our data indicated significant expression alterations of *hsa-miR-30d* and *hsa-let-7b* in NSCLC patients, and that *hsa-miR-30d* could be used to discriminate between NSCLC and nontumor of lung cancer tissue specimens. Based on ROC analysis, it appeared that evaluation of both miRNAs is an informative diagnostic biomarker for lung malignancy. In addition, it seems that downregulations of *hsa-miR-30d* might contribute to tumor initiation and development. Our findings support the potential oncogenic role for *has-let-7b-5p* in NSCLC tumor development.

## References

[1] Hosseini M Naghan PA Karimi S SeyedAlinaghi S Bahadori M Khodadad K Environmental risk factors for lung cancer in Iran: a case-control study. Int J Epidemiol 2009;38(4):989–996. 19589809 10.1093/ije/dyp218

[2] Herbst RS Heymach JV Lippman SM Lung cancer. New Engl J Med 2008;359(13):1367–1380. 18815398 10.1056/NEJMra0802714 PMC10662965

[3] Ettinger DS Akerley W Borghaei H Chang AC Cheney RT Chirieac LR Non-small cell lung cancer. J Natl Compr Canc Netw 2012;10(10):1236–1271. 23054877 10.6004/jnccn.2012.0130

[4] Zhang Y Yang D Weng L Wang L Early lung cancer diagnosis by biosensors. Int J Mol Sci 2013;14(8):15479–15509. 23892596 10.3390/ijms140815479 PMC3759869

[5] Hoffman PC Mauer AM Vokes EE Lung cancer. Lancet 2000;355(9202):479–485. 10841143 10.1016/S0140-6736(00)82038-3

[6] Bartel DP MicroRNAs: genomics, biogenesis, mechanism, and function. Cell 2004;116(2):281–297. 14744438 10.1016/s0092-8674(04)00045-5

[7] Huang Y Shen XJ Zou Q Wang SP Tang SM Zhang GZ Biological functions of microRNAs: a review. J Physiol Biochem 2011;67(1):129–139. 20981514 10.1007/s13105-010-0050-6

[8] Johnson SM Grosshans H Shingara J Byrom M Jarvis R Cheng A RAS is regulated by the let-7 micro-RNA family. Cell 2005;120(5):635–647. 15766527 10.1016/j.cell.2005.01.014

[9] Yu F Yao H Zhu P Zhang X Pan Q Gong C let-7 regulates self renewal and tumorigenicity of breast cancer cells. Cell 2007;131(6):1109–1123. 18083101 10.1016/j.cell.2007.10.054

[10] Akao Y Nakagawa Y Naoe T let-7 microRNA functions as a potential growth suppressor in human colon cancer cells. Biol Pharm Bull 2006;29(5):903–906. 16651716 10.1248/bpb.29.903

[11] Chen D Guo W Qiu Z Wang Q Li Y Liang L MicroRNA-30d-5p inhibits tumour cell proliferation and motility by directly targeting CCNE2 in non-small cell lung cancer. Cancer Lett 2015;362(2):208–217. 25843294 10.1016/j.canlet.2015.03.041

[12] Yao J Liang L Huang S Ding J Tan N Zhao Y MicroRNA-30d promotes tumor invasion and metastasis by targeting Galphai2 in hepatocellular carcinoma. Hepatology 2010;51(3):846–856. 20054866 10.1002/hep.23443

[13] Kumar B Khaleghzadegan S Mears B Hatano K Kudrolli TA Chowdhury WH Identification of miR-30b-3p and miR-30d-5p as direct regulators of androgen receptor signaling in prostate cancer by complementary functional microRNA library screening. Oncotarget 2016;7(45):72593–72607. 27683042 10.18632/oncotarget.12241 PMC5341930

[14] Xuan H Xue W Pan J Sha J Dong B Huang Y Down-regulation of miR-221, -30d, and -15a contributes to pathogenesis of prostate cancer by targeting Bmi-1. Biochemistry (Mosc) 2015;80(3):276–283. 25761682 10.1134/S0006297915030037

[15] Kobayashi N Uemura H Nagahama K Okudela K Furuya M Ino Y Identification of miR-30d as a novel prognostic maker of prostate cancer. Oncotarget 2012;3 (11):1455–1471. 23231923 10.18632/oncotarget.696 PMC3717805

[16] Lu Y Ryan SL Elliott DJ Bignell GR Futreal PA Ellison DW Amplification and overexpression of Hsa-miR-30b, Hsa-miR-30d and KHDRBS3 at 8q24.22-q24.23 in medulloblastoma. PloS One 2009;4(7):e6159. 19584924 10.1371/journal.pone.0006159 PMC2702821

[17] Markou A Tsaroucha EG Kaklamanis L Fotinou M Georgoulias V Lianidou ES Prognostic value of mature microRNA-21 and microRNA-205 overexpression in non-small cell lung cancer by quantitative real-time RT-PCR. Clin Chem 2008;54(10):1696–1704. 18719201 10.1373/clinchem.2007.101741

[18] Zygalaki E Tsaroucha EG Kaklamanis L Lianidou ES Quantitative real-time reverse transcription PCR study of the expression of vascular endothelial growth factor (VEGF) splice variants and VEGF receptors (VEGFR-1 and VEGFR-2) in non small cell lung cancer. Clin Chem 2007;53(8):1433–1439. 17599955 10.1373/clinchem.2007.086819

[19] Cheng HH Yi HS Kim Y Kroh EM Chien JW Eaton KD Plasma processing conditions substantially influence circulating microRNA biomarker levels. PloS One 2013;8(6):e64795. 23762257 10.1371/journal.pone.0064795 PMC3676411

[20] Takamizawa J Konishi H Yanagisawa K Tomida S Osada H Endoh H Reduced expression of the let-7 microRNAs in human lung cancers in association with shortened postoperative survival. Cancer Res 2004;64 (11):3753–3756. 15172979 10.1158/0008-5472.CAN-04-0637

[21] Yanaihara N Caplen N Bowman E Seike M Kumamoto K Yi M Unique microRNA molecular profiles in lung cancer diagnosis and prognosis. Cancer Cell 2006;9(3):189–198. 16530703 10.1016/j.ccr.2006.01.025

[22] Le HB Zhu WY Chen DD He JY Huang YY Liu XG Evaluation of dynamic change of serum miR-21 and miR-24 in pre- and post-operative lung carcinoma patients. Med Oncol 2012;29(5):3190–3197. 22782668 10.1007/s12032-012-0303-z

[23] Markou A Sourvinou I Vorkas PA Yousef GM Lianidou E Clinical evaluation of microRNA expression profiling in non small cell lung cancer. Lung Cancer 2013; 81(3):388–396. 23756108 10.1016/j.lungcan.2013.05.007

[24] Esposito F Tornincasa M Pallante P Federico A Borbone E Pierantoni GM Down-regulation of the miR-25 and miR-30d contributes to the development of anaplastic thyroid carcinoma targeting the polycomb protein EZH2. J Clin Endocrinol Metab 2012;97(5): E710–718. 22399519 10.1210/jc.2011-3068

[25] Zhang P Garnett J Creighton CJ Al Sannaa GA Igram DR Lazar A EZH2-miR-30d-KPNB1 pathway regulates malignant peripheral nerve sheath tumour cell survival and tumourigenesis. J Pathol 2014;232(3):308–318. 24132643 10.1002/path.4294 PMC4166508

[26] Cai JL Liu LL Hu Y Jiang XM Qiu HL Sha AG Polychlorinated biphenyls impair endometrial receptivity in vitro via regulating mir-30d expression and epithelial mesenchymal transition. Toxicology 2016;365:25–34. 27481218 10.1016/j.tox.2016.07.017

[27] Russ R Slack FJ Cigarette-smoke-induced dysregulation of microRNA expression and its role in lung carcino-genesis. Pulm Med 2012;2012:791234. 22191027 10.1155/2012/791234 PMC3236311

[28] Vucic EA Thu KL Pikor LA Enfield KS Yee J English JC Smoking status impacts microRNA mediated prognosis and lung adenocarcinoma biology. BMC Cancer 2014;14:778. 25342220 10.1186/1471-2407-14-778 PMC4216369

[29] Yang X Cai H Liang Y Chen L Wang X Si R Inhibition of c-Myc by let-7b mimic reverses mutidrug resistance in gastric cancer cells. Oncol Rep 2015;33(4): 1723–1730. 25633261 10.3892/or.2015.3757

[30] Yan W Wang S Sun Z Lin Y Sun S Chen J Identification of microRNAs as potential biomarker for gastric cancer by system biological analysis. Biomed Res Int 2014;2014:901428. 24982912 10.1155/2014/901428 PMC4058523

[31] Yu J Feng J Zhi X Tang J Li Z Xu Y Let-7b inhibits cell proliferation, migration, and invasion through targeting Cthrc1 in gastric cancer. Tumour Biol 2015;36 (5):3221–3229. 25510669 10.1007/s13277-014-2950-5

[32] Jukic DM Rao UN Kelly L Skaf JS Drogowski LM Kirkwood JM Microrna profiling analysis of differences between the melanoma of young adults and older adults. J Transl Med 2010;8:27. 20302635 10.1186/1479-5876-8-27 PMC2855523

[33] Fassina A Cappellesso R Fassan M Classification of non-small cell lung carcinoma in transthoracic needle specimens using microRNA expression profiling. Chest 2011;140(5):1305–1311. 21622546 10.1378/chest.11-0708

[34] Yang C Sun C Liang X Xie S Huang J Li D Integrative analysis of microRNA and mRNA expression profiles in non-small-cell lung cancer. Cancer Gene Ther 2016;23(4):90–97. 26964645 10.1038/cgt.2016.5

[35] Ozcan O Kara M Yumrutas O Bozgeyik E Bozgeyik I Celik OI MTUS1 and its targeting miRNAs in colorectal carcinoma: significant associations. Tumour Biol 2016; 37(5):6637–6645. 26643896 10.1007/s13277-015-4550-4

[36] Edmonds MD Eischen CM Differences in miRNA expression in early stage lung adenocarcinomas that did and did not relapse. PloS One 2014;9(7):e101802. 25028925 10.1371/journal.pone.0101802 PMC4100742

[37] Abbasi N Hashemi SM Salehi M Jahani H Mowla SJ Soleimani M Influence of oriented nanofibrous PCL scaffolds on quantitative gene expression during neural differentiation of mouse embryonic stem cells. J Biomed Mater Res A 2016;104(1):155–164. 26255987 10.1002/jbm.a.35551

[38] Jusufovic E Rijavec M Keser D Korosec P Sodja E Iljazovic E Let-7b and miR-126 are down-regulated in tumor tissue and correlate with microvessel density and survival outcomes in non--small--cell lung cancer. PloS One 2012;7(9):e45577. 23029111 10.1371/journal.pone.0045577 PMC3454421

[39] Xu H Liu C Zhang Y Guo X Liu Z Luo Z Let-7b-5p regulates proliferation and apoptosis in multiple myeloma by targeting IGF1R. Acta Biochim Biophys Sin (Shanghai) 2014;46(11):965–972. 25274331 10.1093/abbs/gmu089

[40] Nouraee N Khazaei S Vasei M Razavipour SF Sadeghizadeh M Mowla SJ MicroRNAs contribution in tumor microenvironment of esophageal cancer. Cancer Biomark 2016;16(3):367–376. 26889983 10.3233/CBM-160575 PMC13016487

